# Sequence alignment generation using intermediate sequence search for homology modeling

**DOI:** 10.1016/j.csbj.2020.07.012

**Published:** 2020-07-25

**Authors:** Shuichiro Makigaki, Takashi Ishida

**Affiliations:** Department of Computer Science, School of Computing, Tokyo Institute of Technology Ookayama, Meguro-ku, Tokyo 152–8550, Japan

**Keywords:** Bioinformatics, Homology detection, Homology modeling, Sequence alignment, 62P10, 97M60

## Abstract

Protein tertiary structure is important information in various areas of biological research, however, the experimental cost associated with structure determination is high, and computational prediction methods have been developed to facilitate a more economical approach. Currently, template-based modeling methods are considered to be the most practical because the resulting predicted structures are often accurate, provided an appropriate template protein is available. During the first stage of template-based modeling, sensitive homology detection is essential for accurate structure prediction. However, sufficient structural models cannot always be obtained due to a lack of quality in the sequence alignment generated by a homology detection program. Therefore, an automated method that detects remote homologs accurately and generates appropriate alignments for accurate structure prediction is needed. In this paper, we propose an algorithm for suitable alignment generation using an intermediate sequence search for use with template-based modeling. We used intermediate sequence search for remote homology detection and intermediate sequences for alignment generation of remote homologs. We then evaluated the proposed method by comparing the sensitivity and selectivity of homology detection. Furthermore, based on the accuracy of the predicted structure model, we verify the accuracy of the alignments generated by our method. We demonstrate that our method generates more appropriate alignments for template-based modeling, especially for remote homologs. All source codes are available at https://github.com/shuichiro-makigaki/agora.

## Graphical abstract

**Figure:**
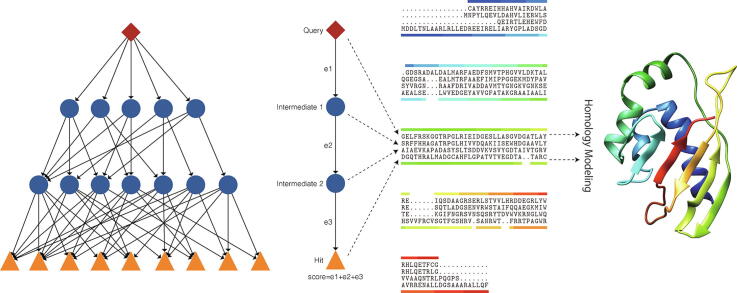

## 1 Introduction

Proteins are key molecules in biology, biochemistry, and pharmaceutical sciences. A protein’s structure often has a strong correlation with its function. Thus, the structure of a protein is an important piece of information in biological research. Protein structures can be determined experimentally using X-ray crystallography or nuclear magnetic resonance spectroscopy, and protein structures determined in this way are registered to, and accessible in the online Protein Databank (PDB) [1]. Despite vast improvements in the available experimental methods for protein structure determination, the speed at which amino acid sequences can be revealed has surpassed our ability to ascertain the corresponding structure. Therefore, protein structure prediction, that is, the use of computational techniques to generate a tertiary structural model of a given amino acid sequence, remains essential.

Homology modeling, or template-based modeling, is one of the computational protein structure prediction methods. It predicts protein structures based on the structure of a template protein and a sequence alignment between the target protein and the template protein. Template structures are the structures of homologous proteins (homologs), and are often found by a homology search of protein structure databases, such as the PDB. Currently, provided one can obtain a good template structure and generate an accurate sequence alignment, template-based modeling is the most practical structure prediction method. This is because the predicted models are more accurate than the other methods, such as *de novo* prediction [2]. However, template-based modeling requires homologous proteins with known structures-to be used as templates. If the protein structure database does not have a homolog entry that closely resembles a query protein, classic sequence homology search algorithms, such as BLAST [3], fail to find a template. Thus, to detect remote (i.e., distantly related) homologs, more sensitive search methods are required. In the first stage of template-based modeling, sensitive and accurate homology detection is essential for accurate structure prediction. In long-term homology detection studies, sequence profiles based on multiple sequence alignments, such as PSI-BLAST [4], DELTA-BLAST [5], and hidden Markov model (HMM)-based methods, representing a category of sequence profile-based methods, can detect remote homologs. In addition, HMM comparison methods, such as HHpred [6], have performed exceptionally well in structure prediction benchmarks [7], [8]. However, even when using the above mentioned sensitive homology search methods, the detection of remote homologs can fail due to insufficient search sensitivity.

To overcome this problem, the intermediate sequence search (ISS) method has been proposed to provide more distantly remote homology detection [9]. The basic idea of ISS is the following: two sequences of remote homologous proteins, which do not have enough sequence identity or a close relationship evolutionally, can be related via another sequence whose characteristics and features are intermediate between the two remotely homologous proteins. If the match score between both the first and third sequences and the second and the third sequences is high, it can be concluded that the first and second sequences are related, even though their sequence similarity is low. In the ISS method, after searching for homologs of the query protein in the database, the results are used as new queries to detect more distantly related homologs by re-running the homology search. By identifying a connection via these intermediate sequences, the ISS method can detect relationships between the original query protein and remote homologs. The idea of the intermediate sequence search itself is not novel [9]. Decades ago, Entrez [10] provided intermediate sequence information. However, the naïve ISS procedure often provides many false positives [11] and requires significant computing resources to evaluate many homology searches. Recently, to overcome the computational demand and occurrence of false positives, approaches that utilize network or graph theory were proposed [12], [13]. In addition, machine learning-based intermediate sequence search methods have demonstrated good results [14], [15].

ISS is a useful technique for improving homology search sensitivity, and several studies have used this method for protein function prediction [16], [11], [9]. However, to our knowledge, there have been no examples of its use in protein structure prediction. The ISS method can detect remote homologs, but it does not generate any sequence alignments between the query and target proteins. As mentioned, template-based modeling requires a template as well as a sequence alignment between the query and template proteins. Thus, to apply template-based modeling to the result of homology detection via ISS, we have to generate a sequence alignment in a separate step. The simplest approach to generate a sequence alignment is through the use of an algorithm, such as Smith-Waterman local alignment [17]. However, it is difficult to generate accurate sequence alignments between remote homologs, and an inaccurate sequence alignment often leads to low quality of predicted models in homology modeling. In essence, alignment quality is crucial to template-based modeling. Thus, in order to apply ISS to template-based modeling, we need a method to generate accurate sequence alignments specifically designed for ISS results.

Sequence alignment generation of remote homologs is a difficult task due to low sequence identity between the query and target sequences. However, for the case of ISS results, we can use additional intermediate sequence information that bridges the two sequences requiring alignment. Thus, we hypothesize that the intermediate sequences would help to generate more accurate sequence alignments.

In this paper, we propose a new sequence alignment generation method for remote homologs detected by an intermediate sequence search for sue in homology modeling. To our knowledge, this is the first study demonstrating the generation and evaluation of alignments using ISS results in the context of template-based modeling. To evaluate the quality of the generated sequence alignments, we performed homology modeling based on the sequence alignments and measured their structure prediction accuracy. As a result, the proposed method showed better accuracy in comparison to a baseline method. Our method is expected to be valuable for distant homologs, and we also evaluate our method for these distant and difficult pairwise targets.

## 2 Materials and methods

**Fig. 1:**
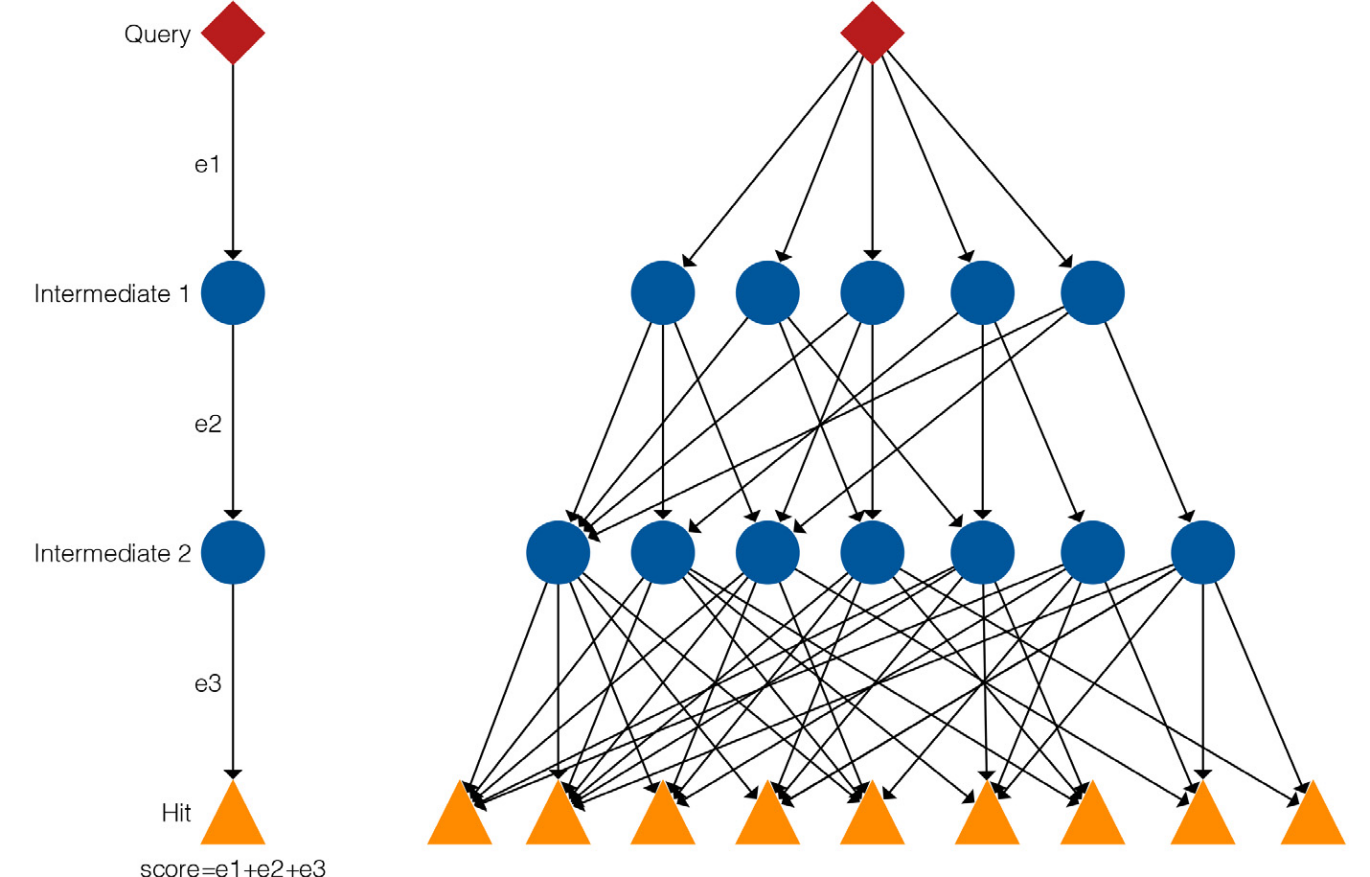
Method overview for intermediate sequence search. Red diamond, blue circles, and yellow triangles show the query, intermediate and hit sequences, respectively. First, we search homologs of a query sequence in the intermediate database and resulting hits represent the first intermediates. Second, these hits are used as queries, such that the next series of find hits represent the second intermediates. Finally, these second intermediates are used as queries again, and we search the final database. Hit score is calculated by the sum of each hit score. (For interpretation of the references to colour in this figure legend, the reader is referred to the web version of this article.)

To detect homologs, we use the intermediate sequence search method. To date, many ISS methods have been proposed to address early problems with the method [12], [13], [14], [15], [16], [11], [9], however, the flow of the searches is basically the same. In this study, we implemented a basic ISS method. The ISS searches for homologs of the query protein in a protein structure or sequence database, and continuously uses the results as new queries to detect more distantly related homologs by re-running the homology search. Fig. 1 shows an overview of the ISS method. As our aim was template-based modeling, the last search had to be done using a structural databases. Any arbitrary databases can be used for the intermediate databases, as they need not be structural databases. To achieve high sensitivity, the intermediate databases should be large; for example, NCBI nr [18] or UniProt [19]. The number of times intermediate searches is one of the parameters in this type of study, and, depending on the level of more sensitivity needed, this can be increased as more depth is required. Also, any search method can be applied for the ISS method. It is recommended that the tools used are fast as execution time often becomes long for numerous intermediate sequences, however, their sensitivity can be low because intermediate sequences should assure sensitivity.

### 2.1 Proposed alignment generation method

Although the detection performance of the ISS is high, this method often provides many false positives [11]. To overcome this problem, we implemented two improvements. First, our method uses a sub-region of the detected sequence as intermediate results to be used as the subsequent query, instead of using the whole sequence of the detected homolog. Many sequences in protein databases consist of multiple domains within one sequence; thus false positives are obtained because the domains, which are not related to the query sequence, are used as subsequent queries in intermediate searches. These domains are inappropriate for remote homology detection and cause many false positives during the ISS [9]. By narrowing the search region to a detected homology region, it is expected that the number of false positives will be reduced. Second, the proposed method assigns rankings to the final results set by the sum of similarities between intermediate sequences. The similarities are calculated during the ISS; for example, if DELTA-BLAST [5] or PSI-BLAST [4] is used, the similarity score will be the Evalue or bit-score. Our method sums similarity scores on the path from query to the final hits, and sorts them to generate the final search results. If multiple paths exist between the query and the final hit, the path with the best one is selected. In this research, we used the Evalue as similarity score and selected a path of the smallest score as the best.

However, it is difficult to generate alignments between remote homologs because the sequence identity between them is often low. Even if some local alignments can be generated, the length of the alignment region is often too short for accurate template-based modeling, such that prediction of the protein structure will not be accurate.

**Fig. 2:**
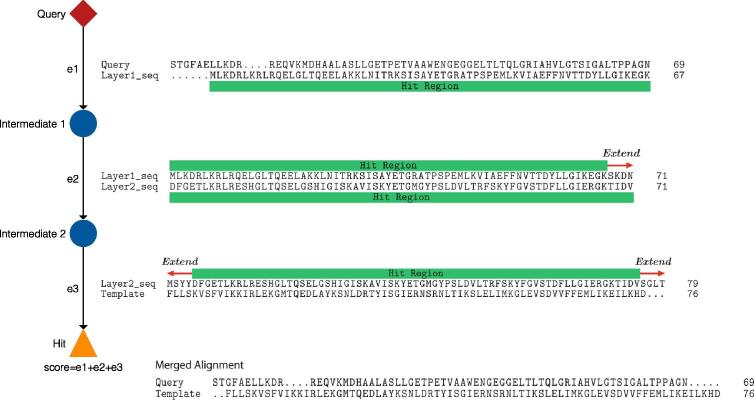
Alignment generation with intermediate sequences. In each intermediate layer, a pairwise alignment is generated. The proposed method merges the pairwise alignments between intermediate sequences. Each sub-pairwise alignment is preserved, which means that the positions of residues in a pairwise alignment are preserved.

To overcome the problem, we use intermediate sequences detected by the ISS. This is reasonable because, in the ISS phase, the proposed method only uses aligned sequence regions, while other domains in a sequence are not used for the search. Fig. 2 shows an overview of our alignment generation method. To extend the aligned region, hit regions are extended in intermediate layers as far as possible such that they do not include other domains too much. The length of the extension is one of the hyperparameters. The extended sub-sequence is used as a query in each intermediate search. In each intermediate layer, pairwise alignments are generated using the Smith-Waterman algorithm.

At the final phase of alignment generation, the proposed method merges pairwise alignments between intermediate sequences. During the merging procedure, no dynamic-programming-based multiple sequence alignment method is used. Each sub-pairwise alignment is preserved, which means that the positions of residues in a pairwise alignment are preserved. A pairwise alignment between the query protein and one of the final hits is then split out from the merged alignment.

**Fig. 3:**
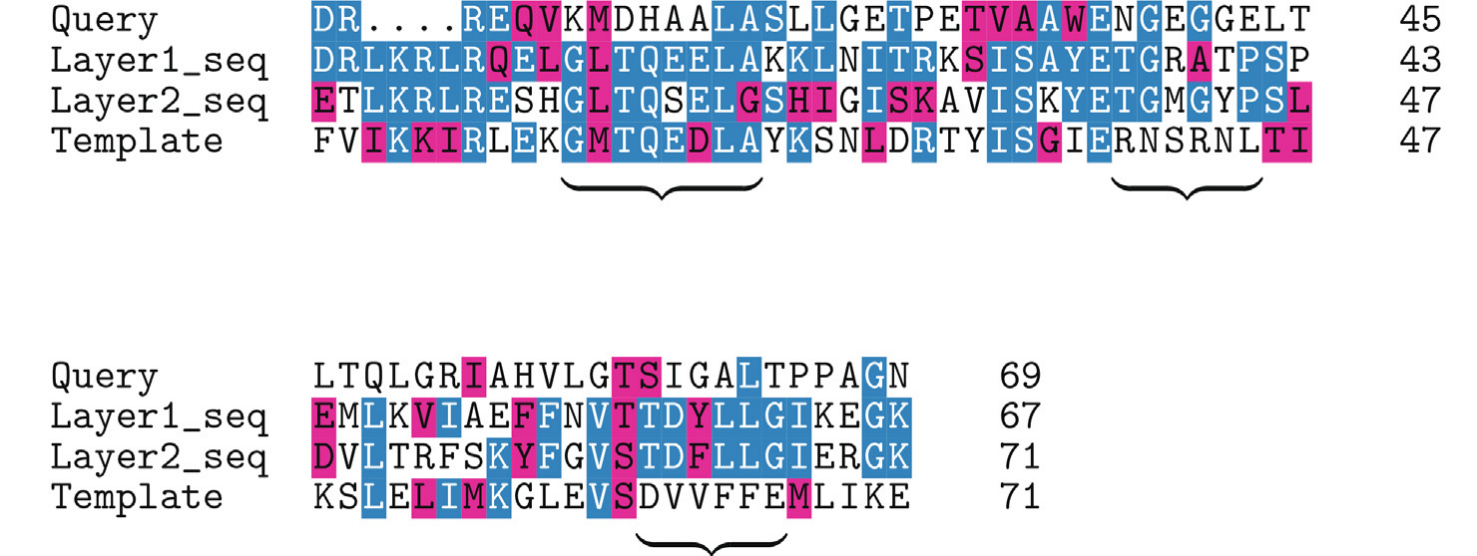
Alignment generation of low a sequence identity region. Sequence similarity of the query and template sequences in the bracketed regions are low. However, the intermediate sub-sequences are similar, and the regions of low sequence similarity are aligned as a result. Blue and red shards identify identical and similar (positive BLOSUM62 score) residues, respectively. (For interpretation of the references to colour in this figure legend, the reader is referred to the web version of this article.)

Fig. 3 shows an example of how intermediate sequences work. Usually, when the pairwise sequence identity of the query and template sequences is low, these regions are not aligned by naïve substitution matrix-based methods. However, if intermediate sub-sequences exist, which are similar to the query or template sequence, similar sequence regions are aligned via these intermediate sequences. These intermediate-proxied alignment regions exist in the merged alignment, and they allow for the extension of aligned regions using remote homologous information.

This alignment method generally produces reasonable alignments, but sometimes generate obviously incorrect alignments because of a large shift of the aligned regions in intermediate search results and so on. Fortunately, we can detect most of these cases by checking the length of the aligned region. Therefore, in addition to merging multiple pairwise alignments, as described above, we also apply the Smith-Waterman algorithm to generate a pairwise alignment of the query and template. Then, we select one that has a longer aligned region.

### 2.2 Materials

In this paper, we used the following datasets for evaluation. UniRef [19] was used as an intermediate sequence database, and Structural Classification of Proteins (SCOP) [20], [21] was used as the final database. The SCOP database classifies proteins by class, folds, superfamily (SF), family, and domain, based on manually curated function/structure classifications. Because the two databases contain redundant sequences, we used UniRef_50_, which was a reduced by clustering sequences of 50% sequence identity; and SCOP_95_ was used as the final database, reduced by 95% sequence identity clustering. DELTA-BLAST was used for the intermediate search tool. For merging intermediate alignments, we used MAFFT’s [22] alignment merge function. For evaluation, we selected 100 sequences for test data from SCOP_40_, which is a SCOP database reduced by 40% sequence identity. Each of the 100 sequences were randomly selected, one from each of the top 100 superfamilies that were sorted according to the size of the superfamily. The selected 100 test domains and their information are listed in Supplementary data.

### 2.3 Evaluation

To evaluate alignment quality for template-based modeling, we generated structural models by template-based modeling from alignments obtained using the proposed method. For template-based modeling, we use the program MODELLER [23]. The TM-score [24] between native protein structure and the predicted one is used as a measure of structure prediction accuracy. The TM-score indicates global structure similarity by a regularized (0,1) value, and a TM-score =1 means the predicted model corresponds to the native structure. We compared the TM-scores of predicted models obtained using the proposed method with two baseline methods. The baseline alignment methods used are the Smith-Waterman algorithm [17], and DELTA-BLAST.

## 3 Results

**Fig. 4:**
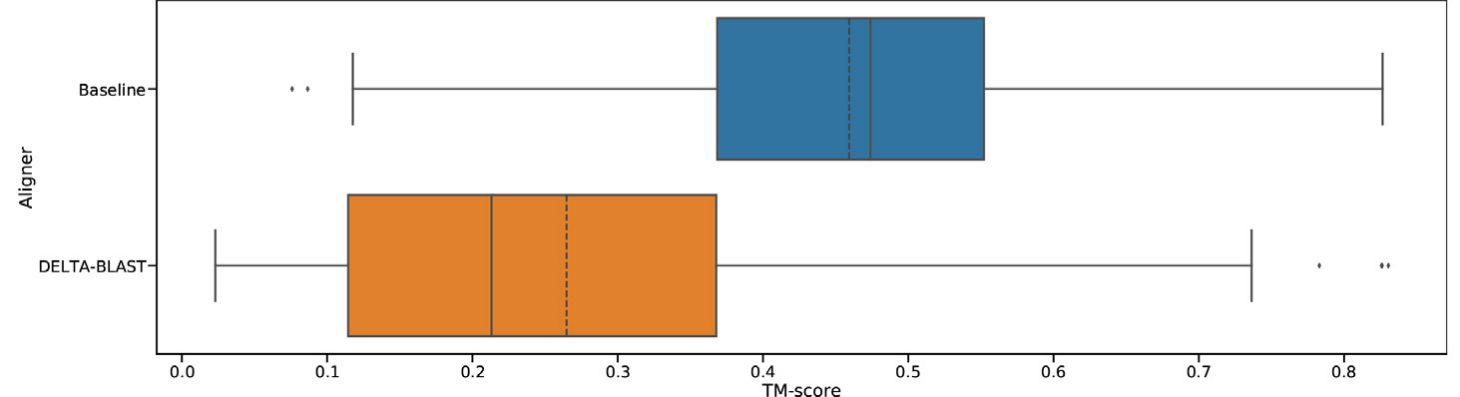
TM-score distribution of hard targets, meaning hits that are not detected by DELTA-BLAST without the use of intermediates. The solid line represents the median, and the dashed line represents the mean. In these results, all templates are from the same superfamily as the query. (For interpretation of the references to colour in this figure legend, the reader is referred to the web version of this article.)

Fig. 4 shows the model accuracy distribution of difficult targets, meaning pairs of query and templates that are not detected using DELTA-BLAST without ISS, but are detected by the proposed ISS method. The figure shows a comparison of the DELTA-BLAST and Smith-Waterman algorithm as a baseline. For DELTA-BLAST, we changed the word score threshold to 1 (-threshold 1) to get longer aligned regions. On average, alignments using DELTA-BLAST did not generate more accurate models than alignments using the Smith-Waterman algorithm.

**Fig. 5:**
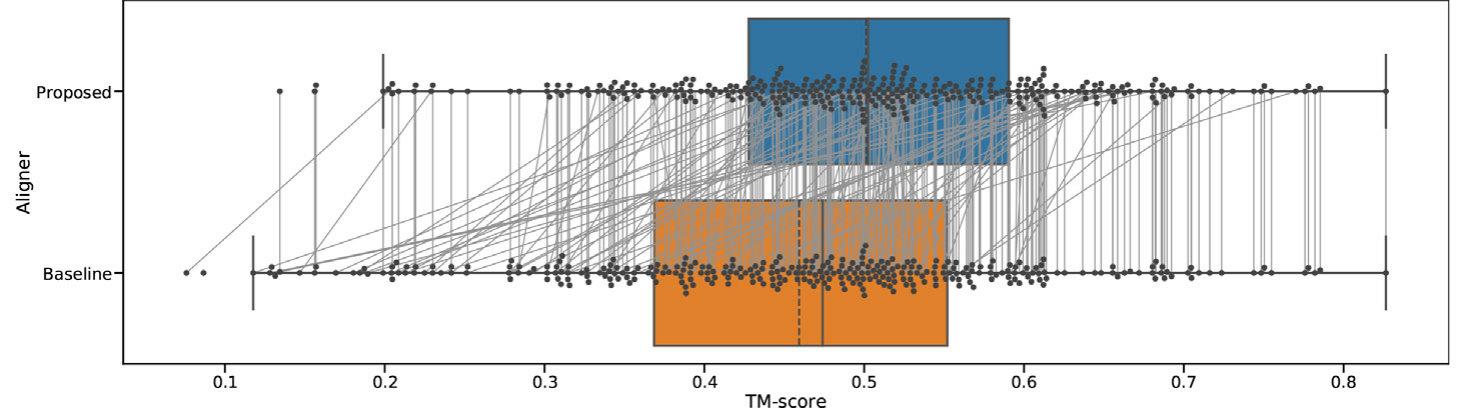
TM-score distribution of hard targets, meaning hits that are not detected by DELTA-BLAST without intermediates. A solid line represents the median, and a dashed line represents the mean. Dots within the boxplot represents individual samples. Gray lines represent the same query and template pair. For the results shown, all templates are from the same superfamily as the query.

Fig. 5 also shows the model accuracy distribution for a difficult target, which is a comparison of the proposed method with the Smith-Waterman algorithm as the baseline. Using these remote homologs, our method generated more accurate models than those generated using the Smith-Waterman alignment, with average TM-scores of 0.50 and 0.46, respectively. We tested the statistical significance using the related *t*-test. The *p*-value was $6.3\times 10^{-13}$, and the average difference is significant (p<0.01). Gray lines indicate the same query and template pair, and they reveal that many models have lower than average TM-scores, and thus improved accuracy.

**Fig. 6:**
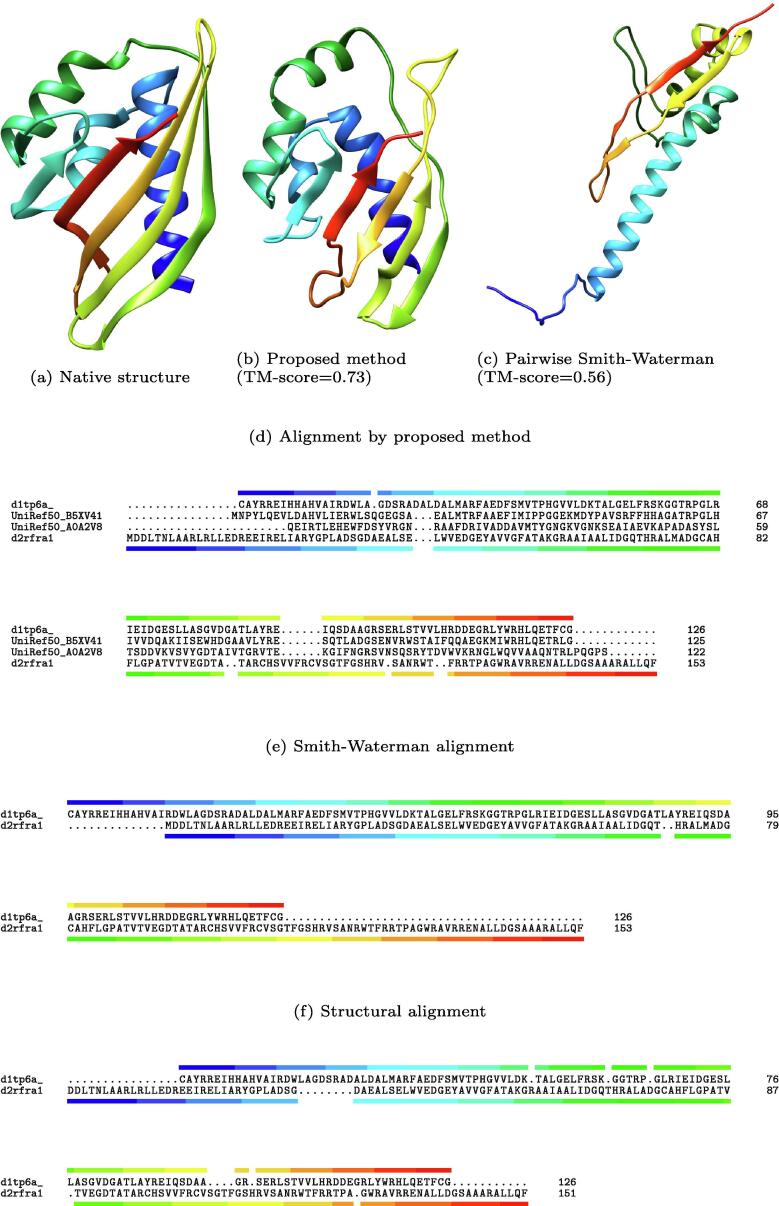
Example 1: SCOP ID of query and template protein are d1tp6a and d2rfra1, respectively.

**Fig. 7:**
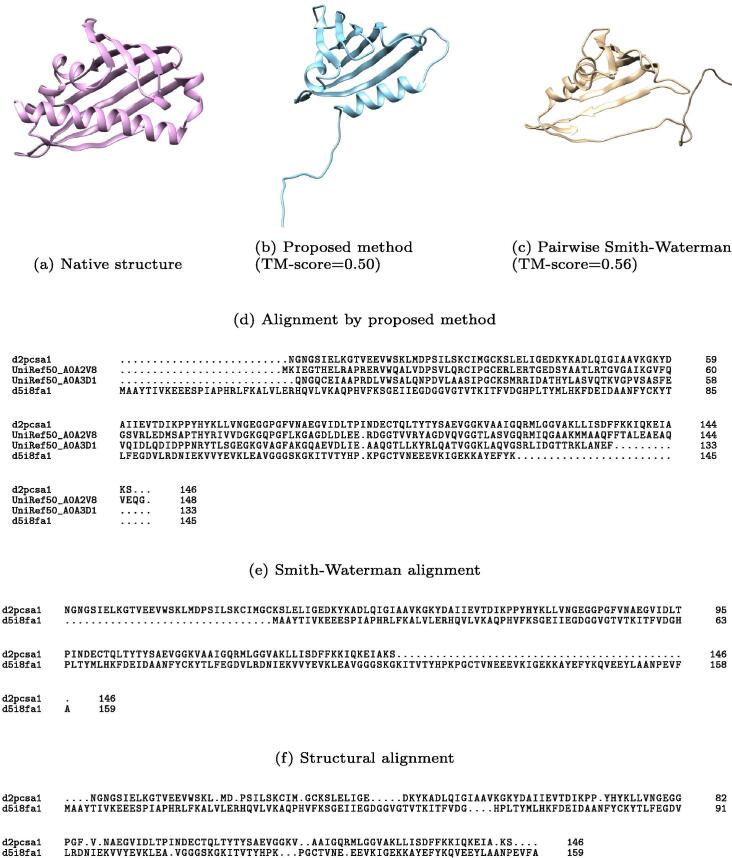
Example 2: SCOP ID of query and template protein are d2pcsa1 and d5i8fa1, respectively.

Fig. 6, Fig. 7 show two examples of results. In these examples, we address alignment quality by comparing them with a structural alignment. In structural alignment, the structural difference between a target protein structure and a template protein structure is minimized; thus, sequence alignments generated by structural alignment are ideal for template-based modeling. Often, the sequence alignments generated by homology detection methods are dissimilar to those generated by structural alignment, especially for remote homologs.

As shown in Fig. 6, the TM-score of the proposed and Smith-Waterman methods are 0.73 and 0.56, respectively. This demonstrates that our method generated a more appropriate alignment for template-based modeling. The TM-score of the query and template is 0.77. The alignment generated using intermediates is similar to the structural alignment. The aligned region of the pairwise Smith-Waterman alignment is right-shifted and narrower in comparison to that obtained using the proposed method. Therefore, the model generated from pairwise the Smith-Waterman alignment is different from the native structure.

In contrast, Fig. 7 shows a slightly worse results than others. The TM-score of the proposed and Smith-Waterman methods are 0.50 and 0.56, respectively. The TM-score of the query and template is 0.75, but the accuracy of the models is approximately 0.5. In this case, structural alignment by TM-align (Fig. 7f) contains many small gap regions, and it is difficult to make a similar alignment using algorithms based on affine-gap. A similar result is obtained when the sequence identity is quite low.

## 4 Discussion

### 4.1 Homology detection accuracy of intermediate sequence search

In this study, we implemented a simple intermediate sequence search to avoid any influence from specific algorithms. Thus, the homology detection accuracy of the search method was unclear. To verify the accuracy of our implementation, we performed an evaluation test using the SCOP database. For the evaluation of detection accuracy, we used a receiver operating characteristic (ROC) curve, because of the imbalance between the number of homologs and non-homologs. For quantitative analysis, we employed the area under the ROC curve (AUC) as the evaluation metrics [25]. Additionally, instead of using the original ROC and AUC, we used ROC_*n*_ and AUC_*n*_ according to previous methods [26]. These methods considered results only up to the *n*th false positive, and AUC_*n*_ was normalized by the number of false positives and cutoff value *n*. We define a true positive homology detection as results that are in the same superfamily as the query protein. By comparing the AUC of homology detection, we evaluated our method against DELTA-BLAST without ISS, as the baseline.

**Fig. 8:**
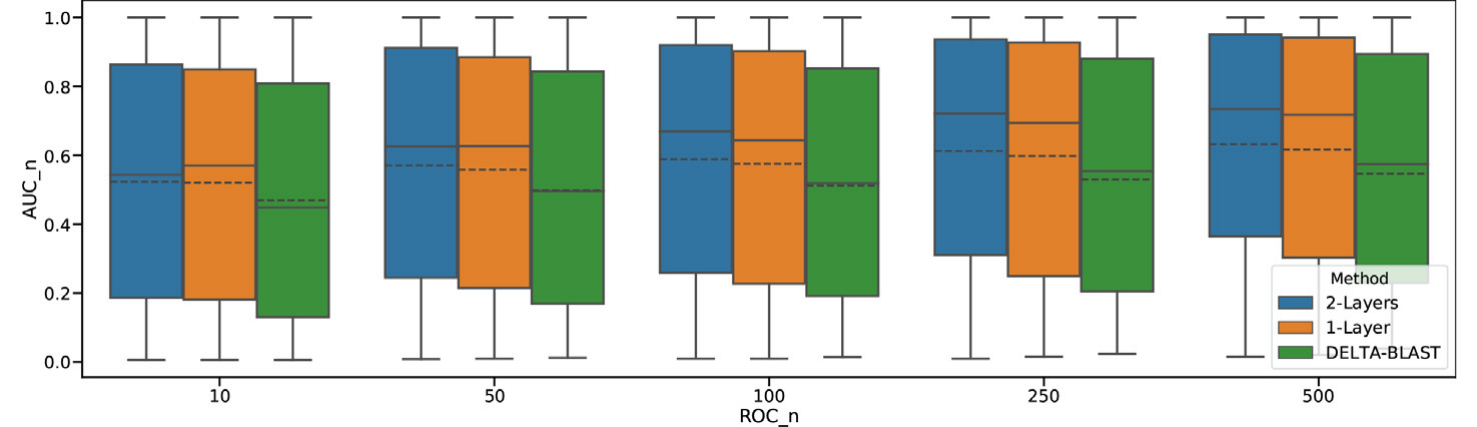
AUC distribution of homology detection. In the boxplots, solid lines indicate medians; dashed lines indicate means. *X* and *Y* axes show the allowed count of false positives and AUC, respectively. 1-Layer and 2-Layers indicate a single intermediate sequence search and double intermediate search, respectively.

Fig. 8 shows the AUC distribution of the accuracy of homology detection. For all of the allowed number of false positives n=(10,50,100,250,500), the proposed method with two intermediate layers overcomes the average AUC of DELTA-BLAST without intermediate layers. In the case of n=10, the average AUC of the proposed method was 0.52 while that of the DELTA-BLAST was 0.46. In the case of n=500, the average of our method reached a value of 0.63 while that of the BLAST approach increased to 0.55. As for the number of intermediate layers, the AUC of two intermediate layers is consistently higher than that of one layer. By increasing the allowed number of false positives (*n*), the AUC of both methods increased.

### 4.2 Model accuracy distribution by various expansion lengths

**Fig. 9:**
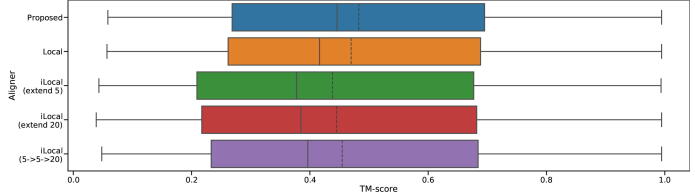
TM-score distribution upon changing extension length. Local and iLocal indicate query-template pairwise Smith-Waterman without intermediates and Smith-Waterman with intermediates, respectively. Solid lines in boxplots indicate median, and dashed lines indicate mean.

Fig. 9 shows the model accuracy distribution upon changing the length of expansion of the hit region. We tried 5 and 20 for the length, and the combination of 5 in intermediate layers and 20 for the final, was used in the proposed method. Longer expansion length could generate more accurate models, and the proposed parameters, which are 5 for two intermediate layers and 20 for the final layer, show the best result. However, query-template pairwise Smith-Waterman alignment shows the highest TM-score average, yet. Finally, our method executes both of Smith-Waterman with intermediates and pairwise Smith-Waterman without intermediates. Using the proposed method, we selected the one that shows a wider aligned region and the results are shown in Fig. 9.

## 5 Conclusion

In this study, we developed an alignment generation algorithm suited for accurate template-based modeling based on intermediate sequence search (ISS) for remote homology detection. Our method used the intermediate sequence search method to detect remote homologs and a sum of similarity score to assign rankings. In the alignment generation phase, we proposed a method that extended the hit region detected by the ISS, and used multiple pairwise alignments between intermediate sequences. In addition to alignment generation, we also applied the pairwise Smith-Waterman algorithm to query and the template sequences, and selected one alignment based on the length of the aligned region. We evaluated our method by comparing the AUC of homology detection for sensitivity and selectivity. We also evaluated the quality of the alignments by comparing the accuracy of template-based structural models generated from the alignments. As a result, the proposed method could detect homologs more accurately than DELTA-BLAST without intermediates. The evaluation of alignment quality based on the accuracy of structural models generated from the alignment, revealed that the proposed method generates more appropriate alignments for template-based modeling, than those prepared without intermediate sequences. As for domains that are not detected by DELTA-BLAST, which were treated as difficult targets, model accuracy measured by TM-score improves by +0.04 on average, when compared using naïve dynamic programming-based alignment.

In this study, we used a simple ISS model to generate alignments using intermediate sequences and evaluate the alignment quality. However, more intelligent ISS methods are available and if used in place of the simple ISS method used here, homology detection performance is expected to improve. Nevertheless, our alignment generation method can be successfully applied to the ISS. Evaluation of the more sophisticated ISS methods remains as one of future work.

## Declaration of Competing Interest

The authors declare that they have no known competing financial interests or personal relationships that could have appeared to influence the work reported in this paper.

## CRediT authorship contribution statement

**Takashi Ishida:** Conceptualization, Resources, Writing - review & editing, Supervision, Project administration, Funding acquisition.
